# The expanding spectrum of neurological disorders of phosphoinositide metabolism

**DOI:** 10.1242/dmm.038174

**Published:** 2019-08-13

**Authors:** Jonathan R. Volpatti, Almundher Al-Maawali, Lindsay Smith, Aqeela Al-Hashim, Julie A. Brill, James J. Dowling

**Affiliations:** 1 Division of Neurology and Program in Genetics and Genome Biology, The Hospital for Sick Children, Toronto, ON M5G 0A4, Canada; 2 Department of Molecular Genetics, University of Toronto, Toronto, ON M5S 1A8, Canada; 3 Department of Genetics, College of Medicine and Health Sciences, Sultan Qaboos University, Muscat 123, Oman; 4 Department of Neuroscience, King Fahad Medical City, Riyadh 11525, Saudi Arabia; 5 Program in Cell Biology, The Hospital for Sick Children, Toronto, ON M5G 0A4, Canada

**Keywords:** ALS, Charcot Marie Tooth disease, Congenital myopathy, Neurogenetic, Phosphoinositides

## Abstract

Phosphoinositides (PIPs) are a ubiquitous group of seven low-abundance phospholipids that play a crucial role in defining localized membrane properties and that regulate myriad cellular processes, including cytoskeletal remodeling, cell signaling cascades, ion channel activity and membrane traffic. PIP homeostasis is tightly regulated by numerous inositol kinases and phosphatases, which phosphorylate and dephosphorylate distinct PIP species. The importance of these phospholipids, and of the enzymes that regulate them, is increasingly being recognized, with the identification of human neurological disorders that are caused by mutations in PIP-modulating enzymes. Genetic disorders of PIP metabolism include forms of epilepsy, neurodegenerative disease, brain malformation syndromes, peripheral neuropathy and congenital myopathy. In this Review, we provide an overview of PIP function and regulation, delineate the disorders associated with mutations in genes that modulate or utilize PIPs, and discuss what is understood about gene function and disease pathogenesis as established through animal models of these diseases.

## Abstract (precis)

**Summary:** This Review highlights the intersection between phosphoinositides and the enzymes that regulate their metabolism, which together are crucial regulators of myriad cellular processes and neurological disorders.

## Introduction

Phosphatidylinositol (PtdIns) and its derivatives are membrane lipids that are composed of a phosphatidic acid (PA) backbone esterified to a myo-inositol ring. This inositol ring can be reversibly phosphorylated at three positions (D3, D4, D5) to generate a group of seven phosphoinositides (PtdIns phosphates or PIPs). PtdIns synthesis begins with the phosphorylation of diacylglycerol (DAG) into PA by DAG kinase. Next, PA is converted into cytidine diphosphate (CDP)-DAG by phosphatidate cytidylyltransferase (Lykidis et al., 1997; D'Souza et al., 2014). The enzyme CDIPT (also known as PtdIns synthase or PIS) then combines CDP-DAG with myo-inositol in the endoplasmic reticulum (ER) to make PtdIns (Lykidis et al., 1997).

**Fig. 1.**
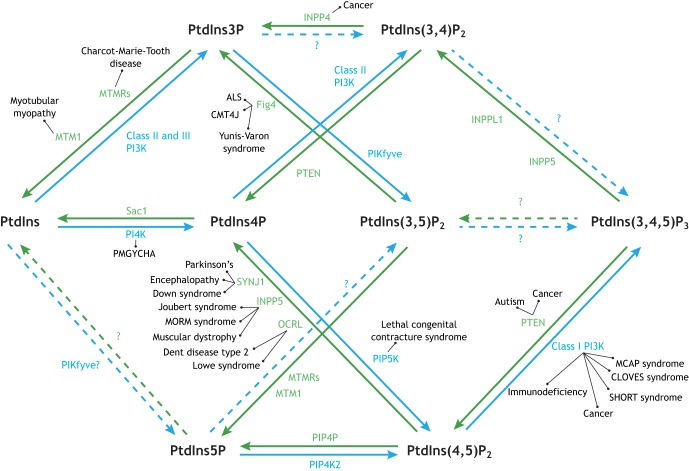
**Phosphoinositide (PIP) metabolism map.** There are seven phosphorylation states of phosphatidylinositol that are targeted by kinases (blue) and phosphatases (green). Perturbations in these pathways are associated with a growing number of neurological disorders, as indicated. Dotted arrows and question marks indicate conversions that have yet to be supported in the literature.

Starting with PtdIns, there are various routes to PIP synthesis that are well described in the literature and we encourage readers to refer to seminal reviews of PIP kinases and phosphatases (Sasaki et al., 2009; Balla, 2013), PIPs and their function (Payrastre et al., 2001; Di Paolo and De Camilli, 2006; Balla, 2013) and PIP effectors (Lemmon, 2008; Kutateladze, 2010) for in-depth discussion. Briefly, PtdIns is reversibly phosphorylated by PtdIns kinases into the PtdIns monophosphates (PtdIns3P and PtdIns4P), which are in turn reversibly phosphorylated to generate PtdIns(3,4)P_2_, PtdIns(3,5)P_2_, and PtdIns(4,5)P_2_ (or PIP2), which can be further phosphorylated to create PtdIns(3,4,5)P_3_ (or PIP3) (Fig. 1). PtdIns5P is unique in that it is primarily generated by dephosphorylation of PtdIns(3,5)P_2_ by 3-phosphatase activity (Fig. 1) (Zolov et al., 2012; Kawasaki et al., 2013; Viaud et al., 2014). It remains unclear whether or not PtdIns5P can be produced directly from PtdIns (Zolov et al., 2012; Sbrissa et al., 2012).

**Fig. 2.**
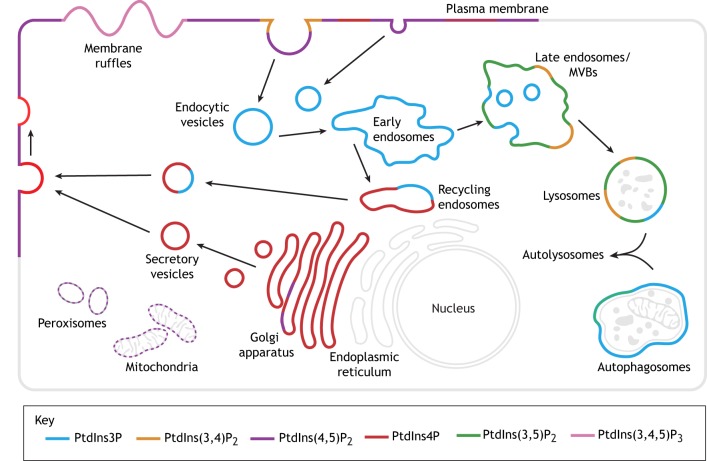
**Subcellular distribution of phosphoinositides.** PIPs confer a unique molecular identity to the membranes of organelles and endocytic compartments, which direct membrane and protein traffic throughout the cell. This figure shows the major pools of PIPs in each compartment but does not exclude that these PIPs are present in other compartments in lower abundance. Please note that PtdIns and PtdIns5P are not shown. MVBs, multivesicular bodies.

Each PIP has a unique subcellular distribution and set of functions that largely overlap with other PIPs, based on their localization and their binding of effector molecules (Fig. 2) (Balla, 2013; Sasaki et al., 2009; Kutateladze, 2010). PIPs regulate numerous cellular processes, including intracellular signaling pathways, cytoskeletal rearrangements, endocytosis, exocytosis, and autophagy (Di Paolo and De Camilli, 2006; Balla, 2013; Sasaki et al., 2009; Kutateladze, 2010; Staiano et al., 2015; Billcliff and Lowe, 2014; Burke, 2018). PIPs also play a critical role in many developmental processes (Skwarek and Boulianne, 2009). Most PIP functions are mediated through PIP-dependent effector proteins (either direct or indirect interactors) (Lemmon, 2008; Kutateladze, 2010), among the most important of which are the Rab family of small GTPases that act as molecular switches to direct membrane fission and fusion events via interactions with a diverse set of Rab-specific effectors (Stenmark and Olkkonen, 2001; Grosshans et al., 2006; Hutagalung and Novick, 2011). PIPs can also serve as second messengers; for example, PtdIns(4,5)P_2_ is hydrolyzed by phospholipase C to form the multifunctional signaling molecules DAG and inositol-3,4,5-trisphosphate (IP_3_) (Payrastre et al., 2001; Di Paolo and De Camilli, 2006).

To improve the understanding of the involvement of PIPs in neurological function, a comprehensive list of genes involved in PtdIns binding and PIP metabolism is presented in Table S1. This list was generated from the existing literature (e.g. Di Paolo and De Camilli, 2006; Sasaki et al., 2009; Balla, 2013 and references therein) and from an InterPro database (https://www.ebi.ac.uk/interpro/) search of genes containing a PIP-binding protein domain (Mitchell et al., 2019). There are 25 PtdIns/PIP kinases (including regulatory subunits), 31 PIP phosphatases and 742 genes with PIP-binding domains. Among these 798 genes, mutations in 163 are associated with a confirmed human genetic disorder as curated in the Online Mendelian Inheritance in Man (OMIM) database (https://www.omim.org/). Intriguingly, 83 of the disease-associated genes feature neurological or neuromuscular involvement, highlighting the importance of PIP regulation in neurological function (Table S2). It is important to note that many genes with a PIP-binding domain in this list have not been functionally validated to determine whether they indeed bind PIPs; thus, this list should serve primarily as a reference for future studies.

In this Review we present the human neurological disorders associated with mutations in PIP-modulating enzymes (Table 1) and in proteins with PIP-binding domains, and discuss the mechanistic relationship between disease and PIP metabolism by focusing on patient-related information and data from cell and animal models. For a more in-depth review of the regulation of PIPs and their involvement in non-neurological disorders, we refer readers to several excellent reviews on this topic (McCrea and De Camilli, 2009; Billcliff and Lowe, 2014; Staiano et al., 2015; Burke, 2018).

**Table:**
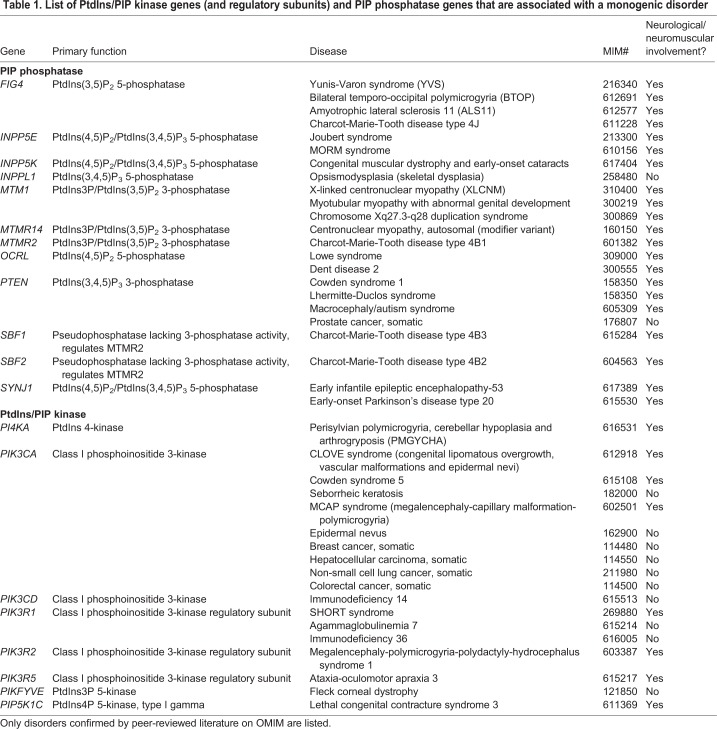
**Table 1. List of PtdIns/PIP kinase genes (and regulatory subunits) and PIP phosphatase genes that are associated with a monogenic disorder**

## Neuromuscular diseases – skeletal myopathies

### Myotubular myopathy

One of the best-characterized neuromuscular disorders of PIP metabolism is the severe childhood muscle disease X-linked myotubular myopathy (XLMTM; MIM 310400) (Laporte et al., 1997; Blondeau et al., 2000; Biancalana et al., 2003; Jungbluth et al., 2008; Lawlor et al., 2016; Dowling et al., 2009; Amburgey et al., 2017). XLMTM is associated with profound muscle weakness that manifests as a failure to achieve motor milestones, wheelchair and ventilator dependence, and death before the age of 10 years in most cases (Amburgey et al., 2017). The disorder is defined by a unique set of skeletal muscle biopsy features that include centrally located nuclei, disorganized perinuclear organelles and myofiber hypotrophy (Dowling et al., 2002; Lawlor et al., 2016). The primary ultrastructural abnormality observed in XLMTM is the disrupted appearance and function of a muscle substructure called a triad (T-tubules and sarcoplasmic reticulum).

XLMTM is caused by mutations in the gene encoding the PtdIns3P phosphatase myotubularin (*MTM1*) (Laporte et al., 1997; Dowling et al., 2002; Biancalana et al., 2003; Amburgey et al., 2017). MTM1 dephosphorylates PtdIns3P and PtdIns(3,5)P_2_ *in vitro*, and regulates endolysosomal maturation and sorting (Blondeau et al., 2000; Tsujita et al., 2004; Velichkova et al., 2010; Ribeiro et al., 2011; Ketel et al., 2016). In muscle tissue, loss of MTM1 is associated with PtdIns3P accumulation (Fetalvero et al., 2013; Pierson et al., 2012; Sabha et al., 2016). The specific interplay between its function as a 3-phosphatase, its ability to modulate endosomal behavior and its essential role in the maintenance of muscle structure remain unknown. This includes the unsolved question of how the regulation of PtdIns3P and PtdIns(3,5)P_2_, which primarily localize to endosomes and lysosomes, respectively, influences the formation and maintenance of the triad, a specialized ER- and plasma membrane-derived structure.

Vertebrate models have provided important insights into how *MTM1* loss causes muscle disease, including the key observation of triad abnormalities. The first model generated was a mouse *Mtm1* knockout (KO) (Buj-Bello et al., 2002), which showed that loss of Mtm1 protein results in muscle pathology, even though the gene is ubiquitously expressed. The *Mtm1* KO mouse has subsequently been instrumental for testing treatment strategies, including the evaluation of adeno-associated virus (AAV)-based gene replacement therapy (Buj-Bello et al., 2008; Childers et al., 2014). It was also used to identify *Pik3c2b*, which encodes a PIP kinase, as a genetic modifier of the disease (Sabha et al., 2016). In mice, *Pik3c2b* KO both prevents and completely reverses the *Mtm1* KO phenotype (Sabha et al., 2016). Interestingly, the knocking out of *Pik3c3*, which encodes another phosphoinositide 3-kinase (PI3K), significantly worsens the *Mtm1* KO phenotype (Sabha et al., 2016). These results suggest that MTM1 and PIK3C2B co-regulate a specific pool of endosomal PtdIns3P that is distinct from that regulated by PIK3C3 (Cao et al., 2007; Gavriilidis et al., 2018). PIK3C3 can generate PtdIns3P at several membrane compartments, including nascent autophagosomal membranes and various intermediates of the endolysosomal pathway (Reifler et al., 2014; Ketel et al., 2016), and conditional KO of *Pik3c3* in skeletal muscle causes lysosomal dysfunction and disrupts autophagy (Reifler et al., 2014). These phenotypes associated with *Pik3c3* KO might explain the worsening of the *Mtm1* phenotype in double mutants, as both zebrafish and mouse MTM1 mutants show impaired autophagic flux (Dowling et al., 2010; Fetalvero et al., 2013).

Two zebrafish models of XLMTM have also been created (Dowling et al., 2009; Sabha et al., 2016). An *mtm1* morphant was instrumental in uncovering the triad abnormalities that are now considered a key pathological change in this disease (Dowling et al., 2009). This model was also used to identify and evaluate drug therapies, such as pyridostigmine, which was shown to have efficacy in the mouse model and is now routinely used in an off-label manner in patients with XLMTM (Dowling et al., 2012). Subsequently, an exon 5 *mtm1* mutant was generated and used to demonstrate that pharmacological and genetic perturbation of specific PI3Ks ameliorates *mtm1* phenotypes (Sabha et al., 2016). There is also a Labrador dog model with an *MTM1* mutation (Beggs et al., 2010). AAV gene replacement therapy has been tested in this model, which has, in turn, provided the critical safety and proof-of-concept data necessary for translation of this therapy to a first-in-human clinical trial (Childers et al., 2014).

### Other centronuclear myopathies

XLMTM belongs to a group of myopathies called centronuclear myopathies (CNM), which are unified by similar features on muscle biopsy (Jungbluth and Gautel, 2014; Ceyhan-Birsoy et al., 2013; Bitoun et al., 2005; Nicot et al., 2007; Nance et al., 2012; Majczenko et al., 2012; Agrawal et al., 2014). Interestingly, several of the genetic causes of CNM affect membrane trafficking and potentially PIP regulation (Bitoun et al., 2005; Nicot et al., 2007; Gonorazky et al., 2018). For example, dominant mutations in dynamin 2 (*DNM2*) and recessive mutations in bridging integrator 1 (*BIN1*) are associated with CNM (Bitoun et al., 2005; Nicot et al., 2007); both proteins physically interact, both have PtdIns(4,5)P_2_-binding motifs, and mutations in both alter their PIP binding (Zhao et al., 2018). In fact, DNM2, BIN1 and MTM1 are all likely to interact (Nicot et al., 2007; Royer et al., 2013). DNM2 levels are increased in XLMTM, and when its levels are reduced in *Mtm1* KO mice, either through germline *Dnm2* mutation or by using antisense oligonucleotides, most aspects of the *Mtm1* KO disease phenotype improve (Cowling et al., 2014; Tasfaout et al., 2018).

A mouse *Bin1* KO mutant has been generated that has a recessive perinatal lethal phenotype and skeletal muscle weakness (Muller et al., 2003). Intriguingly, this severe phenotype is rescued by lowering Dnm2 levels using a similar strategy to that employed for *Dnm2* reduction in *Mtm1* KO mice (Cowling et al., 2017). These data imply that Mtm1 and Bin1 negatively regulate Dnm2 in mice. In addition, *Bin1* overexpression rescues the *Mtm1* KO mouse phenotype (Lionello et al., 2019), further supporting a model of critical interplay and counterbalancing between these three proteins. The exact nature of this interplay is still not understood, nor is it clear how these interactions contribute to the CNM disease process.

Of note, the field is currently hindered by the lack of a pre-clinical model of DNM2-related CNM that phenocopies the clinical aspects of the human disease and thus could be used to develop and test potential therapies. Other models exist, including a mouse *Dnm2* knock-in with a mild phenotype (Buono et al., 2018; Fongy et al., 2019), a zebrafish morphant (Bragato et al., 2016) and a transient dominant *dnm2* zebrafish model (Gibbs et al., 2014), but additional models are clearly needed.

In addition, a very rare form of CNM with cardiomyopathy (found in three unrelated individuals) is caused by recessive mutations in the striated muscle preferentially expressed protein kinase (*SPEG*) gene (Agrawal et al., 2014). SPEG is a serine/threonine kinase that interacts with MTM1 as assessed by a two-hybrid assay (Agrawal et al., 2014) and by co-immunoprecipitation (Agrawal et al., 2014; Gavriilidis et al., 2018). A mouse KO of *Speg* resembles human CNM clinically and histopathologically and has a severe phenotype similar to that of the *Mtm1* KO mouse (Liu et al., 2009). Given the phenotypic similarity between XLMTM and patients with SPEG mutations, and the physical interaction between SPEG and MTM1, it is tempting to speculate that SPEG plays some role in the regulation of PIP metabolism and/or endolysosomal trafficking.

## Neuromuscular diseases – disorders of the peripheral nerve and motor neuron

### Charcot-Marie-Tooth disease

Charcot-Marie-Tooth (CMT) disease is a degenerative disorder of the peripheral nervous system with a global prevalence of 1/2500 (Bird et al., 1998a; Timmerman et al., 2014; Magy et al., 2018). It usually presents as a slowly progressing disease, although age of onset varies from infancy to adulthood and severity can range from mild walking difficulties to a complete inability to ambulate independently (Bird et al., 1998a; Timmerman et al., 2014; Magy et al., 2018). Typical symptoms include distal muscle weakness and sensory loss, distal muscle atrophy and skeletal deformities including, in particular, pes cavus and hammertoes (Bird et al., 1998a).

CMT is categorised into subtypes based on nerve pathology (affecting either the myelinating Schwann cell or the axon that it ensheaths) and mode of inheritance (dominant, recessive or X-linked). Nerve pathology is typically defined by nerve conduction studies: slow conduction velocities signify demyelinating CMT, whereas decreased action potential amplitudes signify axonal CMT (Bird et al., 1998a). Given that CMT is extremely genetically heterogeneous, with causative mutations in more than 80 genes identified to date (Timmerman et al., 2014; Magy et al., 2018), there has been a gradual shift towards a gene-based classification system (Magy et al., 2018). The proteins encoded by CMT genes have diverse cellular and molecular functions, the elucidation of which has also led to seminal insights into the cell biology of the peripheral nervous system (Tazir et al., 2014).

### Charcot-Marie-Tooth disease type 4B

Mutations in genes involved in PtdIns metabolism cause autosomal recessive subtypes of CMT (i.e. CMT type 4; CMT4). These subtypes generally represent severe demyelinating peripheral neuropathies, with onset during early childhood that cause profound disability (Bird et al., 1998b). CMT4B1 (MIM 601382) is caused by mutations in the myotubularin family member *MTMR2* (Bolino et al., 2000), whereas CMT4B2 (MIM 604563), which can also feature glaucoma, is caused by mutations in *MTMR13* (*SBF2*) (Senderek et al., 2003), which encodes a phosphatase-dead myotubularin-related protein that interacts directly with MTMR2 (Robinson and Dixon, 2005). CMT4B3 (MIM 615284), which has a similar presentation to the other two subtypes, is caused by mutations in *MTMR5* (*SBF1*) (Nakhro et al., 2013), which also encodes a catalytically inactive myotubularin family member that interacts with MTMR2 (Kim et al., 2003). These three subtypes share a distinctive nerve pathology, featuring generalized loss of large myelinated nerve fibers and focally folded myelin sheaths (Bird et al., 1998b).

*MTMR2/5/13* are all members of the myotubularin gene family, of which *MTM1* is the canonical member. As with MTM1, MTMR2 is a 3-phosphatase located at the endosome that acts on both PtdIns3P and PtdIns(3,5)P_2_. Its stability and activity are modulated by MTMR5 and MTMR13 (Kim et al., 2003; Robinson and Dixon, 2005). MTMR2 is implicated in endosomal traffic, as is MTM1, although some evidence suggests that MTM1 and MTMR2 act on separate endosomal fractions with different PIP preferences *in vitro* (Cao et al., 2008). That said, when *Mtmr2* is overexpressed in the skeletal muscle of the *Mtm1* KO mouse, it can rescue the KO phenotype (Raess et al., 2017), indicating functional redundancy between these two MTMR family members.

In Schwann cells (the cell type affected in CMT4B), MTMR2 and MTMR13 localize to the cytoplasm and to endomembrane punctae (Ng et al., 2013). The role(s) of MTMR2, MTMR13 and MTMR5 in normal Schwann cell biology, and why their loss causes demyelination, is incompletely understood. In addition, it is not clear why mutations of the two modulators of MTMR2 (MTMR5 and MTMR13) result in the same phenotype and do not exhibit functional compensation.

*Mtmr2*, *Mtmr5* and *Mtmr13* mouse KOs have been generated that phenocopy each other and show initial, early-onset dysmyelination [at postnatal day 3 (P3)] with myelin outfoldings, followed by later nerve degeneration (Bolino et al., 2004; Bonneick et al., 2005; Firestein et al., 2002; Robinson et al., 2008; Tersar et al., 2007). By using these mice, as well as cell culture overexpression, one study has postulated that these proteins regulate epidermal growth factor receptor (EGFR) recycling and thus phospho-Akt signaling (Berger et al., 2011). However, another study that examined peripheral nerve extracts from these KO mutant mice at a different time point did not find evidence of altered Akt signaling (Ng et al., 2013), suggesting a lack of EGF modulation. An alternative hypothesis has been proposed, based on two-hybrid and direct binding assays, that MTMR2 interacts with the scaffold protein discs large 1 (Dlg1) and the motor protein kinesin 13B (Kif13b) (Bolino et al., 2004; Bolis et al., 2009). These studies determined that Dlg1 and Kif13b are mis-expressed and mis-localized in *Mtmr2* KO mice and postulated, based on these molecular interactions and the association of Dlg1 with the exocyst protein Sec8 (Exoc4), that MTMR2 negatively regulates myelin membrane formation, counterbalancing the normal synthesis of myelin.

*Mtmr2* KO mice have been used to identify novel therapeutic targets for CMT4B. Haploinsufficiency of the PIP 5-phosphatase-encoding gene *Fig4*, which is required for both the synthesis and breakdown of PtdIns(3,5)P_2_, lowers PtdIns(3,5)P_2_ levels and ameliorates aspects of the *Mtmr2* KO phenotype in mice (Vaccari et al., 2011). Thus, *Fig4* and *Mtmr2* genetically interact. A recent study (Bolino et al., 2016) has also demonstrated that an inhibitor (apilimod) of the PIP 5-kinase PIKFYVE, which interacts with FIG4 and generates PtdIns(3,5)P_2_, can improve myelination of *Mtmr2* KO-derived Schwann cells *in vitro*. Apilimod appears to be safe in humans (Gayle et al., 2017; Krausz et al., 2012) and future studies aim to assess its efficacy *in vivo* in *Mtmr2* KO mice. Worth noting is the fact that MTMR2 and FIG4, mutations that are discussed extensively in the next section, have a complex relationship. Whereas *Fig4* haploinsufficiency improves the neuropathy phenotype of *Mtmr2* KO mice, *Mtmr2* mutation does not appear to positively modulate the *Fig4* phenotype (and in fact increases motor neuron loss) (Vaccari et al., 2011, 2015).

Another potential treatment strategy has been developed based on the role of neuregulin 1 (Nrg1) type III in regulating myelin formation and the thickness of the myelin sheath. The observation of over-folded myelin (a potentially ‘hypermyelinating’ phenotype) in CMT4B1 led Bolino and colleagues to propose the inhibition of Nrg1 signaling as a treatment (Bolino et al., 2016). They tested this hypothesis using Niaspan, an FDA-approved drug that activates TNF-alpha-converting enzyme, an Nrg1 pathway inhibitor, and found that it rescues the myelin defect observed in *Mtmr2* KO mice (Bolino et al., 2016).

### CMT4J: neuropathy caused by FIG4 mutation

Another subtype of CMT type 4, called CMT4J, is caused by recessive mutations in *FIG4* (also known as *SAC3*) (Chow et al., 2007; Nicholson et al., 2011). CMT4J is characterized by variable age of onset, often with asymmetry in the muscle groups affected, and periods of rapid clinical deterioration (Li et al., 2013). Needle electromyography studies reveal non-uniform slowing of conduction velocities and evidence of denervation (Li et al., 2013). Genetically, patients with CMT4J typically carry one missense change (most commonly the recurrent p.I41T mutation) and one nonsense mutation (Chow et al., 2007; Nicholson et al., 2011). Two *FIG4* nonsense mutations cause a severe neurodegenerative syndrome, Yunis-Varon Syndrome (YVS; MIM 216340) described below (Campeau et al., 2013), with features that are distinct from CMT4J.

FIG4 is a PtdIns(3,5)P_2_-specific 5-phosphatase (Rudge et al., 2004) that exists in a molecular complex with the scaffold protein VAC14 and the 5-kinase PIKFYVE, which together regulate the metabolism of PtdIns(3,5)P_2_ (Gary et al., 2002; Rudge et al., 2004; Jin et al., 2008). Beyond evolutionary explanations, the precise reason that a kinase and phosphatase with opposing roles exist in the same complex is still unknown; however, it is hypothesized that this arrangement enables dynamic spatiotemporally regulated control of PtdIns(3,5)P_2_ (Jin et al., 2008). Much of what we know about FIG4 has emerged from studies in yeast (Gary et al., 2002; Rudge et al., 2004; Jin et al., 2008) and from a spontaneous mouse mutant strain called pale tremor (*plt*) (Chow et al., 2007). The *plt* mouse has an abnormal coat color, manifests a progressive neurodegenerative phenotype that includes limb weakness, spasticity and early death, and extensive vacuolization in the neurons of the central and peripheral nervous system (Chow et al., 2007). The underlying genetic cause of the *plt* phenotype is an insertion mutation at the *Fig4* locus that completely abrogates Fig4 protein production. Because of the striking peripheral nerve phenotype seen in these mice, Meisler and colleagues screened patients with CMT and identified *FIG4* mutations in four unrelated individuals (Chow et al., 2007). Subsequently, numerous CMT patients have been found to have biallelic *FIG4* mutations (i.e. CMT4J).

Loss of *Fig4* in mice is associated with decreased levels of PtdIns(3,5)P_2_ and with slight increases in PtdIns3P, reflecting the role of FIG4 in stabilizing PIKFYVE expression and function. Indeed, a *Pikfyve* hypomorphic mouse with 65-90% reduction in PIKFYVE protein levels across tissues shows moderate to severe neurodegeneration and dies perinatally (Zolov et al., 2012), highlighting the importance of PIKFYVE in neuronal development. Likewise, PIKFYVE likely directly generates all of the PtdIns(3,5)P_2_ in the cell, which is then used to generate the majority of PtdIns5P (Zolov et al., 2012).

The *VAC14* gene encodes the FIG4 and PIKFYVE scaffolding protein VAC14. *Vac14* mutant mice (one KO model and one missense mutant) have a similar phenotype to *plt* mice (Zhang et al., 2007; Jin et al., 2008). Importantly, *Vac14* mutants show reduction in PtdIns(3,5)P_2_ levels (Zhang et al., 2007; Jin et al., 2008). Of note, two patients with a severe neurodegenerative phenotype of progressive debilitating dystonia, loss of motor milestones and deep gray matter changes on brain MRI, characterized as striato-nigral degeneration, childhood-onset (MIM 617054), were reported to have biallelic (compound heterozygous) mutations in *VAC14* (Lenk et al., 2016). In addition, one patient with YVS (discussed below) harbored mutations in *VAC14* (Lines et al., 2017). Taken together, these data suggest that mutations in *FIG4* and *VAC14* result in depletion of PtdIns(3,5)P2 via the loss of PIKFYVE activity (despite the role of FIG4 as a 5-phosphatase) and are associated with similar severe neurodegenerative phenotypes.

On a cellular level, reduction in PtdIns(3,5)P_2_ affects the PIP-sensitive lysosomal calcium channel TRPML1 (MCOLN1; Li et al., 2016). Reduced PtdIns(3,5)P_2_ results in reduced calcium release from TRPML1; this calcium flux is required for the proper maturation of lysosome vesicles, and its reduction is associated with impaired lysosomal motility and tubulation and aberrant autophagy via impaired autophagosome-lysosome fusion (Li et al., 2016). Of note, *TRPML1* mutations result in the disease mucolipidosis type 4 (MIM 252650), which features neuronal vacuolization but has different clinical manifestations compared to *FIG4*-associated CMT4J or YVS (Bargal et al., 2000). TRPML1 agonists have been developed and provide interesting candidates for treating *FIG4*-related disorders. In fact, a chemical agonist that increases calcium flux from TRPML1 can reduce vacuole formation in fibroblasts and in dorsal root ganglia explants derived from *Fig4* mice (Zou et al., 2015). Despite the clear functional interplay between FIG4 and TRPML1, why mutations in these genes cause different phenotypes in not known, though it likely indicates that each gene product has functions beyond lysosomal PtdIns(3,5)P_2_-dependent calcium release via TRPML1.

Atg18p is also part of the FIG4-VAC14-PIKFYVE complex in yeast, acting as a negative regulator (Efe et al., 2007; Jin et al., 2008). The mammalian ATG18 homologs are the WIPI (WD40 interacting with phosphoinositides) proteins. WIPI 1-4 contain WIPI and PROPPIN (β-propellers that bind polyphosphoinositides) domains (Jin et al., 2008) that mediate interaction with PtdIns(3,5)P_2_ and PtdIns3P (Busse et al., 2015). Mutations in two WIPI proteins, WIPI3 (*WDR45B*) and WIPI4 (*WDR45*), cause neurodegenerative disorders (MIM 617977 and MIM 300894, respectively; Table S1) (Haack et al., 2012; Suleiman et al., 2018). This suggests that the loss of regulation of the FIG4-VAC14-PIKFYVE complex could be involved in these disorders.

### Other FIG4-related neurological disorders

Since the discovery of *FIG4* mutations in CMT4J patients, *FIG4* mutations have been associated with other conditions, such as YVS, which is caused by recessive *FIG4* hypomorphic mutations (Nakajima et al., 2013; Campeau et al., 2013). Patients present with prenatal growth retardation and have multiple congenital anomalies, including microcephaly, cataracts, sparse pale hair, facial dysmorphisms and micrognathia (Nakajima et al., 2013; Campeau et al., 2013). Many have congenital heart defects, absent or hypoplastic clavicles, and transverse upper and lower limb anomalies, and most die in infancy (Campeau et al., 2013). Survivors have severe global developmental delay and frequently present brain malformations, including agenesis of the corpus callosum, arhinencephaly, frontal lobe atrophy and pachygyria. On autopsy, cytoplasmic vacuoles that represent endolysosomal bodies are found in the bone, muscle and brain tissue of patients (Campeau et al., 2013).

To date, one family with bilateral temporo-occipital polymicrogyria associated with *FIG4* mutation has been reported (Baulac et al., 2014). The six affected individuals in this family carried a homozygous missense *FIG4* mutation predicted to cause loss of protein function. All presented with complex partial seizures, with onset ranging from birth to 24 years old; two had a psychotic disorder with aggressiveness and delirium, and another two committed suicide. Brain MRI in three patients showed temporo-occipital polymicrogyria. Combined with the MRI changes observed in YVS, these findings suggest that *FIG4* mutations can cause a spectrum of brain malformations potentially associated with impaired neuronal migration.

Lastly, heterozygous *FIG4* mutations have been reported to cause amyotrophic lateral sclerosis (ALS; MIM 612577), a progressive disorder associated with motor neuron pathology (Chow et al., 2009). Nine individuals, six with sporadic and three with familial disease, from a cohort of 473 ALS patients were found to have *FIG4* mutations. The average age of disease onset was 56±14 years. Each had a single, heterozygous *FIG4* mutation, two of which were stop codons, two consensus splice-site variants and five missense mutations; two of the missense mutations were likely pathogenic (Chow et al., 2009). In addition, two *FIG4* missense mutations have been found in a patient with an aggressive form of ALS (Bertolin et al., 2018). However, *FIG4* mutations relatively rarely cause ALS. Other groups did not identify pathogenic mutations in *FIG4* after screening 80 Italian ALS patients (Verdiani et al., 2013), 15 Taiwanese patients with familial ALS (Tsai et al., 2011) and eight ALS families from southeastern China (Liu et al., 2014).

Genotype-phenotype correlations have not been explored in depth for FIG4-related disorders, largely because mutations in FIG4 are rare, particularly outside of CMT4J. One observation is the association between the amount of protein expression and severity, as seen with YVS (caused by biallelic nonsense mutations and thus predicted to result in little or no protein expression) versus CMT4J (in which one mutated allele is typically a missense mutation). Further work is needed to establish genotype-phenotype relationships and, in addition, to define the impact on protein function of missense mutations associated with non-CMT/YVS phenotypes.

## Neurological disorders associated with PtdIns(4,5)P_2_ metabolism

PtdIns(4,5)P_2_ is thought to regulate numerous subcellular processes and signaling cascades. It has been extensively characterized as part of the canonical PtdIns pathway as a substrate for phospholipase C (PLC), producing the second messengers DAG and IP_3_ (Payrastre et al., 2001; Di Paolo and De Camilli, 2006). PtdIns(4,5)P_2_ is also a second messenger, regulating actin filament dynamics and thus important in cell migration, cell-cell adhesion and cytokinesis (van den Bout and Divecha, 2009). It also modulates cell survival, as well as nuclear processes such as cell-cycle progression and splicing (van den Bout and Divecha, 2009). Given its importance, it is perhaps unsurprising that several disorders are associated with abnormalities in PtdIns(4,5)P_2_ regulation.

### Lethal congenital contracture syndromes

Lethal congenital contracture syndromes (LCCSs) are rare conditions associated with severe joint contractures (such as arthrogryposis multiplex congenita), reduced or absent limb movements, and lethality at or soon after birth (Narkis et al., 2007; Koutsopoulos et al., 2013). LCCS3 (MIM 611369) is associated with mutations in *PIP5K1C*. *PIP5K1C* encodes a type I kinase, PIPK1γ, responsible for the synthesis of PtdIns(4,5)P_2_ from PtdIns4P (van den Bout and Divecha, 2009; Lacalle et al., 2015). Narkis and colleagues described two families with LCCS3: one large consanguineous family with nine affected individuals and another family with one affected male (Narkis et al., 2007). Patients had multiple joint contractures with severe muscle wasting and atrophy, but they did not exhibit features seen in other LCCSs, such as hydrops, fractures or multiple pterygia (Narkis et al., 2007). The specific mutation described in both families was the biallelic substitution p.D253N in the PIPK1γ kinase domain that abrogates kinase activity (Narkis et al., 2007).

The hypothesized pathogenic mechanism of *PIP5K1C* mutations concern the known role of PtdIns(4,5)P_2_ in synaptic vesicle trafficking and in clathrin coat dynamics (Di Paolo et al., 2004; Narkis et al., 2007). PtdIns(4,5)P_2_ directly participates in vesicle budding and endocytosis, likely in a dynamin-1-dependent manner (Saheki and De Camilli, 2012). This is in keeping with the phenotype of *Pip5k1c* KO mice, which have decreased PtdIns(4,5)P_2_ levels in the brain and impaired synaptic vesicle dynamics, including slower rates of vesicle endocytosis and recycling (Di Paolo et al., 2004). Another *Pip5k1c* KO mouse manifests neural tube closure defects, cardiac abnormalities and perinatal lethality within the first 24 h after birth and shows reduced ability to produce PtdIns(4,5)P_2_ levels in the brain (Wang et al., 2007).

Interestingly, LCCS5 is caused by recessive mutations in the large GTPase DNM2 (MIM 615368) (Koutsopoulos et al., 2013). This observation might suggest that DNM2 acts in the same pathway as PIPK1γ, given that the pleckstrin homology (PH) domains in dynamin GTPases bind to PtdIns(4,5)P_2_ (Barylko et al., 1998) and dynamins are required for fission of clathrin-coated vesicles (Saheki and De Camilli, 2012). It is plausible that DNM2 uses PtdIns(4,5)P_2_ generated by PIPK1γ to finalize the fission step of clathrin-coated vesicles during synaptic vesicle formation and, when either gene is mutated, synaptic vesicles fail to form and/or recycle efficiently. Future studies should consider systems-based approaches to learn how these genes (and other genes unrelated to PIPs) interact in models of LCCS.

### Neurological ciliopathies related to PIP2 metabolism

Dent disease type 2 and Lowe syndrome (oculocerebrorenal syndrome) are allelic conditions caused by mutations in the X-linked gene *OCRL*, which encodes a PtdIns(4,5)P_2_ 5-phosphatase. Lowe syndrome is characterized by ophthalmological findings, including congenital cataract, glaucoma or microphthalmia, and renal abnormalities, such as proximal tubulopathy, rickets and renal Fanconi syndrome (Lewis et al., 2001). Patients with Lowe syndrome have neonatal hypotonia, and most develop epilepsy and mental retardation (Lewis et al., 2001). The milder form of Lowe syndrome is Dent disease, which manifests primarily with renal findings, including low molecular weight proteinuria and hypercalciuria; patients also occasionally have cognitive and behavioral problems (Lewis et al., 2001; Hichri et al., 2011; Hoopes et al., 2005). Dent disease mutations generally result in a truncated protein that retains some catalytic activity (Shrimpton et al., 2009), whereas Lowe syndrome mutations are frequently characterized by loss of protein expression and/or catalytic activity. Modifier genes might also influence disease severity (Bökenkamp et al., 2009).

Much of our knowledge of OCRL function, as well as of the pathogenic mechanisms caused by *OCRL* mutations, has come from cell and animal models. OCRL localizes primarily to endosomes and the Golgi apparatus (Ungewickell et al., 2004; Vicinanza et al., 2011) as well as to primary cilia in retinal pigment epithelial cells and kidney tubular cells (Luo et al., 2012). Loss of OCRL function is associated with structural and functional abnormalities in the early endosome and with the impaired recycling of multiple receptors (Ungewickell et al., 2004; Vicinanza et al., 2011). Loss of OCRL also results in accumulation of PtdIns(4,5)P_2_ (Zhang et al., 1998), specifically in the transition zone of primary cilia (Prosseda et al., 2017) and in endolysosomes (Festa et al., 2018). PtdIns(4,5)P_2_ accumulation is hypothesized to impair endosomal trafficking to cause disease by increasing endosomal filamentous (F-) actin or by altering F-actin dynamics, which then affects multiple cellular processes (Vicinanza et al., 2011), including cilia formation and function. A transgenic *Ocrl^y/−^;Inpp5b^−/−^* double KO mouse expressing human *INPP5B* was developed as a model of Lowe syndrome and Dent disease (Bothwell et al., 2011) and was used to confirm accumulated PtdIns(4,5)P_2_ in endolysosomes, hyper-polymerization of F-actin and impaired receptor trafficking (Festa et al., 2018).

Interestingly, this transgenic model was developed because the first *Ocrl* KO mouse generated had no obvious phenotype because of functional compensation by INPP5B, another PtdIns(4,5)P_2_ 5-phosphatase (Jänne et al., 1998). This was confirmed via the generation of *Ocrl/Inpp5b* double KO mice, which have a lethal embryonic phenotype (Jänne et al., 1998). Such compensation appears to depend on tissue-specific expression patterns of *OCRL* and *INPP5B* (Bernard and Nussbaum, 2010). Indeed, Luo and colleagues observed that *OCRL* and *INPP5B* have differential co-expression patterns between human and mouse eye tissue (Luo et al., 2013). Furthermore, they show that *ocrl* and *inpp5b* act synergistically in primary ciliogenesis, suggesting genetic compensation in zebrafish (Luo et al., 2013). Collectively, these data illustrate the possibility that *OCRL*-related disorders could be rescued by increasing levels of INPP5B, perhaps via AAV gene delivery or via drugs known to upregulate *INPP5B* expression in Lowe syndrome-affected tissues.

In zebrafish, mutation of *ocrl* causes a phenotype with many of the human disease features, including cystic brain lesions, seizures and kidney abnormalities (Ramirez et al., 2012). This model was subsequently found to have mild defects in cilia formation and impaired endocytic recycling (Oltrabella et al., 2015). Intriguingly, the *ocrl* mutants accumulate PtdIns(4,5)P_2_, which can be rescued through morpholino knockdown of the PIP 5-kinase *pip5k1ab*, suggesting that PIP5K could also be a therapeutic target for Lowe and Dent syndromes (Oltrabella et al., 2015).

Mutations in *INPP5E*, which encodes a PtdIns(4,5)P_2_ and PtdIns(3,4,5)P_3_ 5-phosphatase, can cause Joubert syndrome (MIM 213300) and MORM syndrome (which stands for mental retardation, truncal obesity, retinal dystrophy and micropenis; MIM 610156) (Bielas et al., 2009; Jacoby et al., 2009). Both conditions are autosomal recessive and feature abnormal cilia formation, and are thus classed as ciliopathies. Joubert syndrome is characterized by cerebellar hypoplasia and abnormal brain stem development (Parisi and Glass, 2003; Valente et al., 2005). It is usually accompanied by developmental delay, atypical eye movements and a dysregulated breathing pattern, which causes episodic tachypnea or apnea. Patients can also present with retinal dystrophy, renal disease, ocular colobomas, occipital encephalocele, hepatic fibrosis, polydactyly, oral hamartomas and endocrine abnormalities (Parisi and Glass, 2003). MORM syndrome is milder and features moderate mental retardation from early childhood (Hampshire et al., 2006).

The *INPP5E* mutations that cause Joubert syndrome diminish the 5-phosphatase activity of the protein (Bielas et al., 2009), whereas MORM syndrome mutations result in a protein with phosphatase activity but that abnormally localizes to cilia (Jacoby et al., 2009). *Inpp5e* homozygous null mice are perinatal lethal and display typical signs of a ciliopathy, including bilateral anophthalmos, postaxial hexadactyly and polycystic kidneys, as well as cerebral developmental defects, including anencephaly and exencephaly (Jacoby et al., 2009). Primary cilia from the renal cysts of these KO mice are abnormal in size and morphology (Jacoby et al., 2009).

When *Inpp5e* is conditionally inactivated in the mouse kidney epithelium, its loss causes severe polycystic kidney disease (Hakim et al., 2016) due to the loss of INPP5E 5-phosphatase-mediated hydrolysis of PtdIns(3,4,5)P_3_, which results in increased pAkt and the activation of the mTOR growth pathway. When mTOR was inhibited in this study with everolimus, the size and weight of kidneys in the *Inpp5e* conditional KO mouse were reduced. This corresponded with a 50% improvement in renal function compared to placebo, indicating that the combination of mTOR inhibition and PI3 kinase inhibition (to reduce pAkt) could be the most effective means of rescuing this phenotype.

Insights into the spatiotemporal regulation of PIPs by INPP5E have also shed light on the dynamic process that controls ciliogenesis. In non-ciliated cells, INPP5E localizes at the basal body and maintains a PtdIns4P-high, PtdIns(4,5)P_2_-low environment. INPP5E-produced PtdIns4P controls the recruitment of Tau-tubulin kinase 2 (TTBK2) to the mother centriole, inhibiting its interaction with the centrosomal protein CEP164. In the absence of INPP5E, the PtdIns4P 5-kinase PIPKIγ consumes PtdIns4P to make PtdIns(4,5)P_2_, coinciding with the initiation of ciliary elongation (Xu et al., 2016). In ciliated cells, Chávez and colleagues and Garcia-Gonzalo and colleagues independently showed that INPP5E depletes cilia of PtdIns(4,5)P_2_ to maintain a PtdIns4P-high environment (Chávez et al., 2015; Garcia-Gonzalo et al., 2015). Upon loss of INPP5E, PtdIns(4,5)P_2_ accumulates in cilia, leading to the recruitment of GPR161 and the PtdIns(4,5)P_2_-binding protein Tubby-like protein 3 (TULP3), which negatively regulate sonic hedgehog (SHH) signaling (Chávez et al., 2015; Garcia-Gonzalo et al., 2015).

Consistent with these findings, Dyson and colleagues recently demonstrated that expression of constitutively active smoothened (Smo) re-activated the SHH pathway in *Inpp5e* KO mice, rescuing many phenotypes (Dyson et al., 2017). Conduit and colleagues (Conduit et al., 2017) expanded on these findings, showing that INPP5E hydrolyzes PtdIns(3,4,5)P_3_ in the transition zone (TZ) of cilia, thereby decreasing pAkt inhibition of pGSKβ, promoting both the ciliogenesis and SHH signaling pathways. When INPP5E 5-phosphatase activity is lost, PtdIns(3,4,5)P_3_ accumulates in the TZ, resulting in increased pAKT inhibition of pGSKβ, leading to cilia loss. Given that cilia are required for proper SHH signaling, INPP5E loss disrupts the progression of SHH-dependent medulloblastoma (Conduit et al., 2017). Importantly, PI3K inhibition prevents PtdIns(3,4,5)P_3_ accumulation and restores TZ components to the cilia (Dyson et al., 2017). Thus, although PI3K inhibition is a potential therapeutic target for ciliopathy in Joubert syndrome, it could have a detrimental effect in the context of SHH-driven medulloblastomas by sustaining ciliogenesis and driving proliferation.

### Parkinson's disease type 20

Early-onset Parkinson's disease type 20 (MIM 615530) is caused by biallelic mutations in synaptojanin 1 (*SYNJ1*), which encodes a PtdIns(4,5)P_2_/PtdIns(3,4,5)P_3_ 5-phosphatase (Quadri et al., 2013; Krebs et al., 2013). Affected individuals have a neurological phenotype with Parkinson's disease features (Quadri et al., 2013; Krebs et al., 2013). Treatment with L-DOPA is effective but causes dyskinesia. Early-onset generalized seizures that pre-dated the Parkinsonism were reported in one family (Quadri et al., 2013). In addition, two affected members in another family showed cognitive decline in their thirties and forties, with their brain MRIs showing diffuse cerebral cortical atrophy (Krebs et al., 2013).

It is important to note that synaptojanin is highly expressed in the brain, as compared to OCRL, which is ubiquitously expressed in all tissues. Synaptojanin 1 also contains a polyphosphatase SAC1-like domain that is essential for synaptic vesicle endocytosis in neurons and is thus essential for the maintenance of neuronal transmission (Clayton et al., 2013). *Synj1* KO mice die shortly after birth and exhibit numerous neurological defects, including severe weakness, ataxia and convulsions (Cremona et al., 1999; Clayton et al., 2013). Recently, Fasano and colleagues showed that SYNJ1 regulates the morphology of early endosomes (EE) and plays an important role in trafficking and recycling of proteins through the EE compartment of neuronal cells (Fasano et al., 2018). The authors propose that defects in the endocytic compartment provide insight into the mechanisms governing altered neuronal plasticity and/or the loss of neuronal viability seen in Parkinson's disease type 20.

Interestingly, mutations in *SYNJ1* also result in a subtype of early infantile epileptic encephalopathy (EIEE; MIM 617389). The mouse phenotype thus appears to recapitulate the parkinsonian and epileptic features of human disease. In addition, in mouse models of Down syndrome, SYNJ1 overexpression contributed to enlarged EE and brain dysfunction (Cossec et al., 2012). This finding makes SYNJ1 an interesting potential target for the treatment of Down syndrome (Cossec et al., 2012).

### Congenital muscular dystrophy and early-onset cataracts

Several reports have linked biallelic mutations in *INPP5K* to congenital muscular dystrophy with cataracts and intellectual disability (MIM 617404). INPP5K is a PtdIns(4,5)P_2_/PtdIns(3,4,5)P_3_ 5-phosphatase, preferring PtdIns(4,5)P_2_ as a substrate (Dong et al., 2018). Wiessner and colleagues reported on eight families with 12 affected individuals with hypotonia during infancy, cataract onset between six months and six years of age, and delayed motor development (Wiessner et al., 2017). These patients also had a mild intellectual disability but were without facial or oculomotor weakness or cardiac involvement. Creatine kinase levels were high in affected individuals, between four to fourteen times the upper limit of normal. And nine patients had non-specific muscle pathology, showing variable degrees of dystrophic features. In three patients, muscle biopsies also showed vacuolated muscle fibers. The four *INPP5K* mutations reported in these eight families were all missense mutations that did not appear to affect protein level upon overexpression studies in COS-7 cells; however, phosphatase activity was reduced (Wiessner et al., 2017).

Osborn and colleagues independently reported on four additional families with five affected individuals with biallelic *INPP5K* mutations (Osborn et al., 2017). They all presented during infancy or in early childhood with muscle weakness and motor delay, and exhibited moderate to severe intellectual disability. Cataracts were noted in three patients, and brain MRI showed normal cerebellum and white matter. Creatine kinase was consistently high (>1000 U/L). The reported mutations included four missense that were confirmed to reduce the phosphatase catalytic activity of INPP5K. However, the C-terminal frameshift deletion p.Asn417Lysfs*26, which is located outside of the phosphatase domain and reported in one patient, did not significantly affect the catalytic activity; protein levels in patients or cell models were not investigated (Osborn et al., 2017).

INPP5K dysfunction has been modeled in zebrafish using morpholino-mediated knockdown of one or both zebrafish paralogs (*inpp5ka* and *inpp5kb*) (Osborn et al., 2017). The resulting *inpp5ka* morphants showed microphthalmia, microcephaly, curled and shortened bodies, severely impaired swimming and an abnormal touch-evoked escape response. Histological findings in skeletal muscle showed abnormal myosepta, gross myofiber disorganization and reduced neuromuscular junction arborization, suggestive of poor synaptic formation. Electron microscopy revealed less compact myofibrils, short sarcomeres, as well as undefined A- and I-bands, and reduced triad size. A recent study has uncovered a role for INPP5K in the regulation of ER morphology whereupon loss of INPP5K results in expansion of ER sheets (Dong et al., 2018). It remains to be determined whether this mechanism is involved in the onset of this form of congenital muscular dystrophy; however, it is an intriguing possibility given the importance of specialized ER, i.e. sarcoplasmic reticulum, in skeletal muscle function. As for the intellectual disabilities seen in patients, Dong and colleagues point out that these are consistent with the function of INPP5K in neurons (Dong et al., 2018).

### Disorders of PIP acyl chain composition

An example of neurologic disease related indirectly to PtdIns(4,5)P_2_ comes from *MBOAT7* mutations, which cause autosomal recessive mental retardation 57 (MIM 617188). *MBOAT7* encodes a lysophosphatidylinositol acyltransferase (LPIAT) that remodels the fatty acid composition of PtdIns in a process called the Lands cycle (Lee et al., 2008, 2012). *Mboat7* null mice die prematurely and display a smaller cerebral cortex and hippocampus with lamination defects (Lee et al., 2012). This study further showed that loss of LPIAT activity reduces the amount of polyunsaturated fatty acid, such as arachidonic acid, that is incorporated into the *sn*-2 position of PtdIns, increasing the saturated fatty acid composition in phosphorylated derivatives, such as PtdIns4P and PtdIns(4,5)P_2_ (Lee et al., 2012). This study highlights that fatty acid composition might influence PIP localization and/or their interaction with PIP-binding proteins. An improved understanding of how changing the fatty acid composition of PIPs can modify models of PIP disorders will further our understanding of PIP biology and perhaps lead to the discovery of novel targets for therapeutic intervention.

## PI4K-related disorders

Dysregulation of the mono-phosphorylated PIP PtdIns4P is also implicated in neurologic disease. Mutations in the *PI4KA* gene, which encodes a PI4 kinase type IIIα, cause PMGYCHA (MIM 616531), a disorder characterized by bilateral perisylvian polymicrogyria, cerebellar hypoplasia, arthrogryposis and variable contractures (Pagnamenta et al., 2015). PI4KA generates PtdIns4P from PtdIns, and PtdIns4P serves as a precursor to maintain plasma membrane PtdIns(4,5)P_2_ levels (Bojjireddy et al., 2014; Tan et al., 2014). A recent report has provided insight into the pathogenic mechanisms underlying the PI4KA-related central nervous system abnormalities. When Alvarez-Prats and colleagues specifically deleted *Pi4ka* from Schwann cells in mice, the resulting mutants had severe myelination defects (Alvarez-Prats et al., 2018). It also resulted in the depletion of plasma membrane PtdIns4P, but no decrease in PtdIns(4,5)P_2_. However, plasma membrane phosphatidylserine, phosphatidylethanolamine and sphingomyelin were significantly decreased as well, suggesting that the regulation of PtdIns4P by PI4KA is important for maintaining levels of other lipid species at the plasma membrane, which are essential for the proper myelination of Schwann cells. It remains unclear, however, how impaired PI4KA alters brain development and neuronal migration in order to cause polymicrogyria.

Interestingly, a mouse KO for type II PI4K, *Pi4k2a*, develops late-onset features resembling hereditary spastic paraplegia (HSP), with severe axonal degeneration in the spinal cord that is associated with tremors, high-frequency nodding, spastic gait, limb weakness and urinary incontinence (Simons et al., 2009). Several genes associated with HSP are related to axon transport, and the phenotype of *Pi4k2a* mice is consistent with defective axon transport. Indeed, the inhibition of PI4K activity disrupts the retrograde axonal transport of neurotrophins, and the acute effects of PI4KIIa knockdown or inhibition include disrupted intracellular trafficking and phosphoinositide signaling (Bartlett et al., 2002; Minogue, 2006). Given the importance of PIPs in membrane trafficking, their involvement in HSP-like pathology is unsurprising. The effects of *Pi4k2a* deficiency in mice may result from a direct deficit of PtdIns4P or its PtdIns(4,5)P_2_ derivative. Nevertheless, as yet, no *PI4K2A* mutations have been found in patients with HSP, so the relevance of these findings to human disease is unknown.

## Neurological disorders of genes with PIP-binding domains

All PIP metabolic disorders can be considered, at least in part, to be disorders of PIP-binding proteins, given that the loss of PIP kinases and phosphatases is associated with changes in absolute and local PIP levels, thereby disrupting the localization and functions of myriad PIP-binding effectors. There are as many as 70 neurological disorders that result from mutations in genes containing a PIP-binding domain (Table S1); here, we present a few notable examples that provide insights into the function of specific PIPs in disease.

Loss-of-function mutations in C2 domain-containing phospholipase C beta 1 (*PLCB1*), which cleaves PtdIns(4,5)P_2_ into DAG and IP_3_ (McLaughlin et al., 2002), and in PH domain-containing dynamin 1 (*DNM1*), which binds to PtdIns(4,5)P_2_ during membrane fission events (Barylko et al., 1998), cause two similar subtypes of EIEE (MIM 613722 and MIM 616346, respectively) (Kurian et al., 2010; EuroEPINOMICS-RES Consortium et al., 2014). The EEIE phenotype is also associated with mutations in *SYNJ1*, as discussed earlier. The similar presentations might reflect a common pathway involving these genes in the etiology of EIEE. Indeed, a large-scale survey of causative mutations in EIEE suggested synaptic dysregulation as a common underlying mechanism (EuroEPINOMICS-RES Consortium et al., 2014); however, this hypothesis has yet to be functionally validated for the majority of these genes. This disorder might also provide a unique example of how a disease of a PIP-metabolizing enzyme causes abnormal PIP-binding effector activity, or vice versa. It is worth mentioning here that another PH domain-containing protein that binds PtdIns3P, collybistin (*ARHGEF9*), is also mutated in EIEE (Alber et al., 2017; Wang et al., 2018). However, patients with missense mutations in the PH domain do not develop seizures, whereas patients with missense mutations affecting other regions of the gene have epilepsy (Alber et al., 2017; Wang et al., 2018), suggesting that PIP binding may not have a critical role in the manifestation of this subtype of EIEE.

Mutations in dysferlin (*DYSF*) present as Miyoshi muscular dystrophy (MIM 254130) and limb-girdle muscular dystrophy type 2B (MIM 253601) (Weiler et al., 1996; Liu et al., 1998). Their common features include proximal muscle weakness and dystrophic features on skeletal muscle biopsy (Weiler et al., 1996; Liu et al., 1998). Dysferlin has been implicated in sarcolemmal repair and T-tubule biogenesis. Dysferlin contains seven C2 domains, of which only the N-terminal-most domain C2A, binds to phosphatidylserine, PtdIns4P and PtdIns(4,5)P_2_ in a calcium-dependent manner (Therrien et al., 2009). It has recently been shown that dysferlin-mediated plasma membrane tubulation requires binding to PtdIns(4,5)P_2_ (Hofhuis et al., 2017), further linking PtdIns(4,5)P_2_ (which can be bound by DNM2 and BIN1) to T-tubule generation and skeletal muscle function and disease.

Lastly, mutations in two genes called protrudin (*ZFYVE27*) and spastizin (*ZFYVE26*), which encode FYVE domain-containing proteins, cause subtypes of spastic paraplegia (MIM 610244 and MIM 270700, respectively) (Mannan et al., 2006; Hanein et al., 2008). Common features include axonal degeneration, progressive lower-limb spasticity and weakness of the lower limbs (Mannan et al., 2006; Hanein et al., 2008). Defective endolysosomal processes and autophagy have been implicated in both subtypes (Khundadze et al., 2013; Vantaggiato et al., 2013; Renvoisé et al., 2014; Raiborg et al., 2015; Hong et al., 2017), suggesting these proteins have roles in a common pathway.

Enlarged LAMP-1 membrane compartments were observed in brain tissue from a *Zfyve26* mouse KO (Khundadze et al., 2013) as well as in patient-derived fibroblasts (Renvoisé et al., 2014). Consistent with this observation, reduced lysosome-autophagosome fusion and accumulation of autophagosomes was observed in spastizin-mutant fibroblasts (Vantaggiato et al., 2013). Spastizin colocalizes with the early endosome marker EEA1 and ER markers (Hanein et al., 2008; Khundadze et al., 2013) and physically interacts with beclin 1 in the endosomal PIK3C3 complex II (Vantaggiato et al., 2013), linking its function with autophagy induction.

Protrudin localizes to the ER, where it mediates ER-to-lysosome and late endosome (LyLE) contact sites by binding to PtdIns3P on LyLEs (Raiborg et al., 2015; Hong et al., 2017). This contact allows transfer of kinesin-1 motor proteins to the LyLEs, which translocates mTOR- and synaptotagmin VII-containing LyLEs along microtubules to the cell periphery (Raiborg et al., 2015; Hong et al., 2017). This maintains mTOR in its activated state to attenuate autophagy and also promotes fusion with the plasma membrane to promote neurite outgrowth (Raiborg et al., 2015; Hong et al., 2017). Interestingly, mutations in kinesin-1 (*KIF5A*) also cause a spastic paraplegia (MIM 604187) (Ebbing et al., 2008), as do mutations in the gene encoding the microtubule-severing protein spastin (*SPAST*) (MIM 182601) (Svenson et al., 2001; Roll-Mecak and Vale, 2008), consistent with the proposed mechanism of action for protrudin by Raiborg and colleagues (Raiborg et al., 2015). This is a potential example of how a common pathway can mechanistically unify genetically heterogeneous disorders. It will thus be interesting to explore potential crosstalk between spastizin and the protrudin pathway given that they regulate similar autophagic processes and may involve a common pool of PtdIns3P.

## PI3K/Akt-related conditions

PI3K signaling via Akt is involved in many overgrowth syndromes and cancers (Fresno Vara et al., 2004; Fruman and Rommel, 2014; Mayer and Arteaga, 2016). Class I PI3Ks, such as PIK3CA, phosphorylate the inositol ring of PtdIns(4,5)P_2_ at the 3-position to generate PtdIns(3,4,5)P_3_. The pathways activated by PtdIns(3,4,5)P_3_ and PtdIns(3,4)P_2_ control various functions, including growth and proliferation. In the PI3K/Akt pathway, the 3-phosphatase PTEN acts as a negative regulator of PI3K signaling owing to its ability to hydrolyze PtdIns(3,4,5)P_3_ (Song et al., 2012). Of note, PTEN is one of the most frequently mutated tumor suppressors found in cancer (Fruman and Rommel, 2014). In addition to its role in cancer, loss of function causes PTEN hamartoma tumor syndromes such as Cowden syndrome 1 (MIM 158350) (Blumenthal and Dennis, 2008). This group of syndromes (Table 1) is notable for macrocephaly, benign and malignant tumors, abnormal skin manifestations such as lipomas, and pigmented macules (Blumenthal and Dennis, 2008). In addition, PTEN mutations are associated with macrocephaly/autism syndrome (MIM 605309) (Butler et al., 2005).

In recent years, there has been increasing recognition of the role of somatic mutations in certain neurodevelopmental syndromes. Somatic mutations occur in several diseases associated with epilepsy, autism spectrum disorders and intellectual disability (Poduri et al., 2013). Recent advances in the genomic sequencing and bioinformatic analyses of these variants has allowed us to more accurately estimate the contributions of such phenomena to human neurological disorders (Evrony et al., 2016).

Mutations in the PI3K/Akt pathway are known to cause somatic neurologic syndromes. Postzygotic activating mosaic mutations of *PIK3CA* have been associated with overgrowth syndromes, including MCAP (megalencephaly-capillary malformation-polymicrogyria syndrome; MIM 603387) (Rivière et al., 2012) and CLOVES syndrome (congenital lipomatous overgrowth, vascular malformations and epidermal nevi; MIM 612918) (Kurek et al., 2012). Both conditions are characterized by increased birth weight and height, asymmetric limb hemihypertrophy and vascular malformations. Patients with MCAP also manifest megalencephaly, brain malformation, significant developmental delay and seizures, whereas CLOVES patients have significant skeletal abnormalities, such as megaspondylodysplasia, or hyperostosis of the skull, in addition to skin manifestations of lipomas or linear epidermal nevus (Kurek et al., 2012; Lindhurst et al., 2012; Rivière et al., 2012). Recently, 19 patients with CLOVES were treated with the PIK3CA inhibitor BYL719 and all showed marked improvement in disease symptoms (Venot et al., 2018). Germline mutations of the PI3K regulatory subunit *PIK3R2*, as well as the predominant AKT serine/threonine kinase isoform in the brain *AKT3*, also cause MCAP (Rivière et al., 2012).

Of note, somatically acquired mutations of *PIK3CA* are reported in many cancers, including breast (MIM 114480), colorectal (MIM 114500), gastric (MIM 613659), hepatocellular (MIM 114550) and ovarian (MIM 167000) (Table 1). The importance of PI3K in cancer is underscored by the anticancer effect of drugs that inhibit downstream effectors of PI3K/Akt signaling, such as rapamycin, an mTOR inhibitor (Yuan and Cantley, 2008). There are many excellent reviews that discuss in detail the role of the PI3K/Akt pathway and mTOR in cancers and neurodevelopmental disorders (Fresno Vara et al., 2004; Fruman and Rommel, 2014; Mayer and Arteaga, 2016).

## Conclusion

A growing number of neurological disorders are associated with mutations in genes that encode PIP-metabolizing enzymes and PIP-binding proteins, underscoring the importance of these low-abundance phospholipids for health and disease. Studying these diseases, particularly in cell and animal models, has identified crucially important roles for PIPs and the enzymes that regulate them in organismal biology, including their participation in key processes, such as synaptic vesicle recycling, skeletal muscle T-tubule biogenesis and maintenance, peripheral myelination, and cilia formation and signaling. These studies have also unveiled new therapeutic avenues and treatments for several neurological disorders.

Nevertheless, there is still much to learn about the regulation of PIPs in many neurological disorders. PIPs define organelle identity, acting as molecular signposts for many effector proteins and molecular switches, such as Rab GTPases, to coordinate membrane and protein traffic throughout the cell (Di Paolo and De Camilli, 2006; Ketel et al., 2016). Given this role in regulating distinct processes via effector proteins, PIPs can be regarded as being pleiotropic in nature. This pleiotropy poses one of the major challenges in the field of PIP disorders: determining the primary causal mechanism(s) of disease by demonstrating a causal relationship between one or multiple processes that are regulated by the PIP and the onset of the disorder. For example, we know that loss of MTM1 in XLMTM results in PtdIns3P accumulation, leading to aberrant receptor trafficking and recycling through endosomal compartments (Velichkova et al., 2010; Ribeiro et al., 2011; Sabha et al., 2016; Ketel et al., 2016), and that these changes correlate with disease severity. However, we still do not know how many PtdIns3P effector(s) behave differently in this disease, or how many protein(s) are trafficked improperly, and whether or not these changes cause the observed disease phenotype. To complicate matters, this assumes that the sole function of MTM1 is to dephosphorylate PtdIns3P, which is certainly not the case given its phosphatase-independent functions (Amoasii et al., 2012) and role in the ubiquitin-proteasome system (Gavriilidis et al., 2018).

**Fig. 3.**
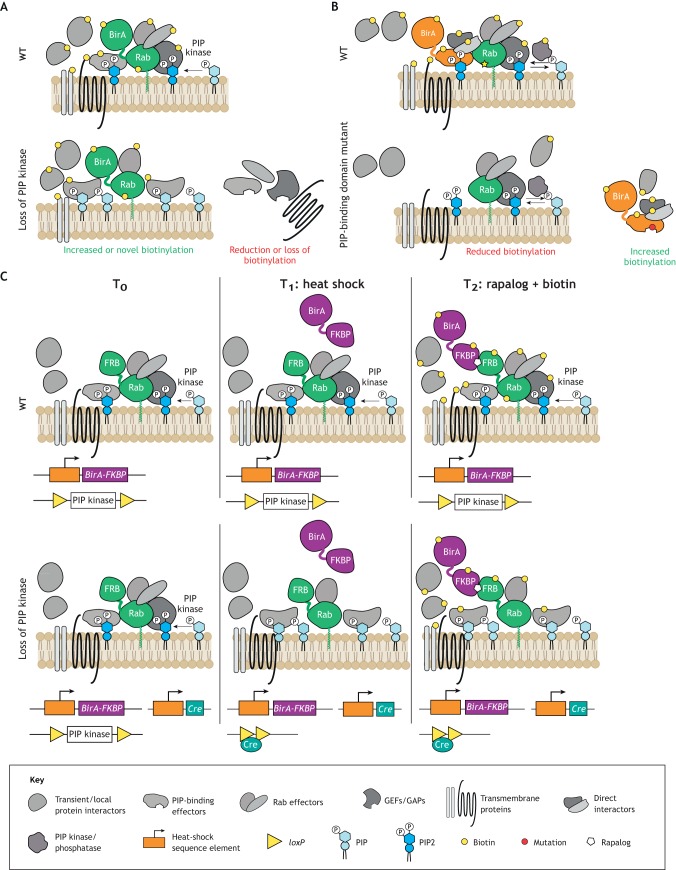
**Potential experimental approaches to interrogate pleiotropy in phosphoinositide biology.** PIPs and their effectors have pleiotropic functions. Elucidating these will require methods that capture global changes that occur when PIPs are altered. This figure shows the complex protein networks that interact with PIPs and illustrates the possible combination of BioID screening (Roux et al., 2013) with inducible protein expression/localization methods to investigate how PIP kinase mutants or PIP-binding protein mutants acquire differential subcellular protein interactomes. (A) A Rab GTPase can be used as an organelle marker that can be fused with the biotin protein ligase BirA, which biotinylates nearby endogenous PIP-interacting proteins, to provide subcellular specificity to a differential interactome. This interactome, mapped based on biotinylation (yellow circle), is different between wild-type cells (upper panel) and cells that do not express, for example, a PIP kinase (lower panel). (B) If BirA is fused to a PIP-binding protein, mutations in a PIP-binding protein (red circle) alter the interactome. This results in a BioID screening output showing biotinylation of PIP-independent interactors, whereas the PIP-containing compartment will lose or have reduced biotinylation. Although useful as an organelle marker, fusing Rab to BirA may result in BioID screens identifying non-specific transient interactions, such as those with the Rab GDP dissociation inhibitor GDI. These methods could be extended upon with artificial regulation of protein complex formation, such as the rapamycin- or rapalog-induced dimerization between FKBP- and FRB-fused proteins (Inobe and Nukina, 2016), and with inducible gene editing to increase spatiotemporal control. (C) Concomitant heat-shock-induced expression of the Rab-FRB fusion, the BirA-FKBP fusion and Cre recombinase, and addition of a rapalog to induce FKBP-FRB dimerization, allows for simultaneous control over protein biotinylation and Cre-induced deletion of a PIP kinase. This would allow for identification of differential subcellular protein interactomes before and shortly after knockout of a gene (in this example, a PIP kinase).

One potentially universal strategy to elucidate PIP-dependent membrane and protein traffic processes would be to perform subcellular proteomics in inducible KO models. Subcellular proteomics can be performed in a variety of ways, including, but not limited to, techniques such as TurboID (and its predecessor BioID; Fig. 3A,B) (Branon et al., 2018; Roux et al., 2013) or subcellular fractionation profiling followed by mass spectrometry (Borner et al., 2014; Itzhak et al., 2016; Christoforou et al., 2016). Inducible KOs can also be achieved in a variety of ways, such as tamoxifen- or tetracycline-driven and heat-shock-inducible Cre/Lox recombination (Fig. 3C) (Imai et al., 2001; Bäckman et al., 2009; Le et al., 2007). Increasingly sophisticated methods of gene perturbation with modified CRISPR/Cas9 technologies allow for rapid and tunable gene editing (Senturk et al., 2017). The combination of these methods would allow researchers to determine the spatiotemporal changes in local proteomes that occur before and after a given PIP is modulated (by kinases or phosphatases; Fig. 3) or when a given PIP-binding protein is lost. In keeping with the MTM1 example, this would involve performing proteomics on specific endosomal populations, myonuclei and/or the sarcolemma, before and after MTM1 is inducibly knocked out. This should, hypothetically, provide rich information about how PtdIns3P [or PtdIns(3,5)P_2_] effectors behave when MTM1 is lost, and a detailed timeline of how pathologic changes begin.

Alternatively, a powerful system that facilitates spatiotemporal manipulation of PIPs was first published over a decade ago by two independent groups (Varnai et al., 2006; Fili et al., 2006). This system uses rapamycin (or its analogs, rapalogs) to recruit PIP enzymes fused to the FKBP12-rapamycin binding (FRB) domain of mTOR [or the rapamycin intracellular receptor FK506 binding protein (FKBP)] to specific subcellular compartments labeled with a well-established marker fused to FKBP (or FRB) via FRB-FKBP heterodimerization (Fig. 3C) (Inobe and Nukina, 2016). This system has since been used to temporally manipulate PIPs, in particular PtdIns(4,5)P_2_ and PtdIns4P, with great success (Szentpetery et al., 2010; Ufret-Vincenty et al., 2015; Gulyás et al., 2017). However, this technique still awaits widespread adoption, particularly to study PIP metabolism disorders. Future studies interested in directly linking PIP metabolism or PIP binding by an effector protein to a disease mechanism may benefit from adopting this powerful yet underutilized approach.

Applying unbiased techniques that capture a global view of the protein localization changes that occur when a PIP is modulated would undoubtedly improve our understanding of PIP biology and its role in disease. We also recommend considering more bioinformatic, systems biology approaches to identify a common pathway for genes that cause the same disorder because, as we have learned, many neurological disorders are genetically heterogeneous. Earlier, we presented an elegant example by Raiborg and colleagues in which they implicated several genes that cause spastic paraplegia in a common pathway (Raiborg et al., 2015). Similarly, the identification of *DNM2* and *BIN1* as genetic modulators of *MTM1* (Cowling et al., 2017; Lionello et al., 2019), all of which cause a centronuclear myopathy, indicates intimate crosstalk among genes involved in the same (or similar) disorders. We recommend that future research on genetically heterogeneous disorders (e.g. Charcot-Marie-Tooth syndromes, epileptic encephalopathies, spinocerebellar ataxias) consider this theme when trying to build a mechanistic model of disease pathogenesis.

On this note, it is worthwhile mentioning how the disruption of other PtdIns derivatives results in neurological diseases. Mutations in PtdIns glycan anchor biosynthesis (PIG) genes such as *PIGP* (MIM 617599) and *PIGA* (MIM 300868) can cause early infantile epileptic encephalopathy (Table 2). Other glycosylphosphatidylinositol (GPI) synthesis genes are associated with severe cognitive impairment and seizures: *PIGM* mutations cause glycosylphosphatidylinositol deficiency (MIM 610293), those in *PIGL* cause CHIME syndrome (MIM 280000), *PIGY* mutations cause hyperphosphatasia with mental retardation syndrome 6 (MIM 616809), whereas those in post-GPI attachment to proteins 1 (*PGAP1*) cause mental retardation 42 (MIM 615802) (Table 2). Most recently, Nguyen and colleagues showed individuals with mutations in *GPAA1*, a GPI transamidase complex protein, present with developmental delay, hypotonia, early-onset seizures, cerebellar atrophy and osteopenia (MIM 617810) (Nguyen et al., 2017).

**Table 2.**
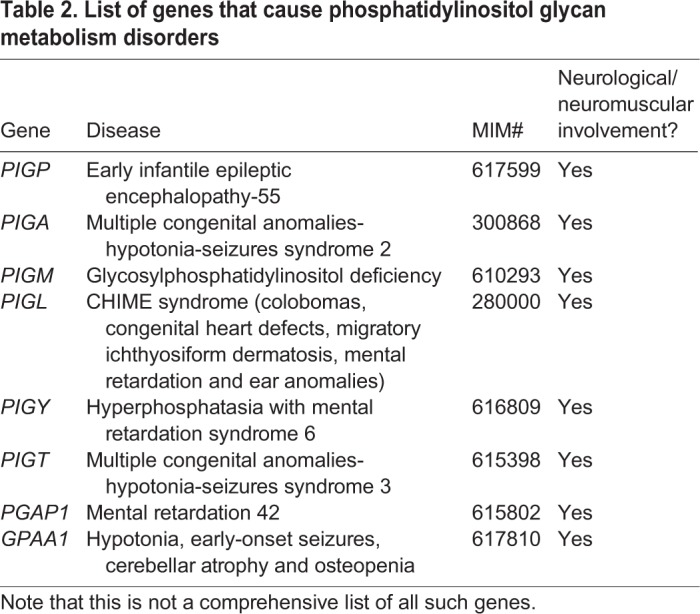
**List of genes that cause phosphatidylinositol glycan metabolism disorders**

Given that many PtdIns/PIP kinases and PIP phosphatases phenotypically overlap in these conditions, it is tempting to infer an intimate crosstalk between the phosphorylated and glycoslyated derivatives of PtdIns. Indeed, mutations in *PLCB1*, *DNM1*, *ARHGEF9* and *SYNJ1* cause EIEE, and mutations in *MBOAT7* as well as 13 genes with PIP-binding domains (*ARHGEF6*, *CASK*, *CC2D1A*, *CNKSR2*, *COL4A3BP*, *DLG3*, *EPB41L1*, *FGD1*, *IQSEC2*, *KIF1A*, *OPHN1*, *SYNGAP1*, *TRIO*) cause mental retardation (Table S1). It is possible that a glycosylation defect in PtdIns impinges on PIP-related pathways given that GPI proteins anchor approximately one in every 200 mammalian proteins to the cell membrane (Nguyen et al., 2017). These examples further emphasize the global importance of PtdIns metabolism in normal brain development and represent additional areas for future research.

With the more widespread application of techniques, such as whole-exome sequencing in clinical genetics, it is unsurprising that new associations between human diseases and mutations in PIP enzymes are emerging. Still, as many as 636 PIP-modulating or -binding proteins have not, as of yet, been linked with human genetic disorders (Table S1), indicating that they are either indispensable for early embryonic development or functionally redundant. Lastly, because of their enzymatic properties, kinase and phosphatase regulators of PIP metabolism are attractive therapeutic targets for neurological disease. This has been demonstrated for human cancers, for which PI3 kinase inhibitors have long been considered as potential treatments. In the future, inhibitors or activators of the PIP enzymes and PIP-binding proteins associated with neurological disease are likely to emerge as viable therapeutics.

## Supplementary Material

Supplementary information
