# Supplementary material for: The expanding spectrum of neurological disorders of phosphoinositide metabolism
Source: Dis Model Mech. 2019 Aug 13;12(8):dmm038174. doi: 10.1242/dmm.038174 (PMC6737944; doi:10.1242/dmm.038174)
Supplement: Supplementary information [file dmm-12-038174-s1.pdf]

Table S1. Comprehensive list of PtdIns/PIP kinase genes (and regulatory subunits), PIP phosphatase genes, and genes containing PIP-binding domains that are associated with a monogenic disorder. Only disorders with confirmed (by peer-reviewed literature) or with provisional status on OMIM are listed. Genes with PIP-binding domains were sourced from InterPro.

Click here to Download Table S1

Table S2. List of Mendelian disorders and list of genes encoding PIP-modulating genes (i.e. PIP kinases, PIP phosphatases, and proteins with PIP-binding domains).

Click here to Download Table S2

Disease Models & Mechanisms • Supplementary information
